# Impact of Residual Glutaraldehyde-Didecyldimethylammonium Bromide on Fermentation Performance and Microbial Community in Ectopic Fermentation System

**DOI:** 10.3390/microorganisms14102136

**Published:** 2026-09-22

**Authors:** Zhuomacairang Wang, Richeng Cui, Jiayin Tong, Pengliang Liu, Shuntao Huang, Yiren Gu, Gan Luo

**Affiliations:** 1 College of Animal and Veterinary Sciences, Southwest Minzu University, Chengdu 610041, China; 2 Key Laboratory of Qinghai-Tibetan Plateau Animal Genetic Resource Reservation and Utilization, Ministry of Education, Southwest Minzu University, Chengdu 610041, China; 3 Key Laboratory for Animal Science of National Ethnic Affairs Commission, Southwest Minzu University, Chengdu 610041, China; 4 Buffalo Research Institute, Chinese Academy of Agricultural Sciences and Guangxi Zhuang Nationality Autonomous Region, Nanning 530001, China

**Keywords:** pig manure slurry, ectopic fermentation system, disinfectants residue, bacterial community, metagenomics

## Abstract

Ectopic fermentation systems (EFSs) are widely applied to treat livestock manure, yet residual disinfectants transferred from production areas into the manure collection system may compromise their performance, and the consequences of such continuous low-dose input remain unclear. Here, we aimed to determine whether long-term input of the widely used disinfectant glutaraldehyde–didecyldimethylammonium bromide (GD) impairs EFS operation, and to identify the changes in microbial communities and organic matter underlying these effects. Pig manure slurry containing 20 mg/kg GD was supplied continuously, and physicochemical parameters, enzyme activities, microbial communities, antibiotic resistance genes (ARGs), mobile genetic elements (MGEs), and three-dimensional excitation–emission matrix (3D-EEM) spectra were monitored throughout the fermentation period. GD significantly reduced total nitrogen accumulation (*p* < 0.05) and suppressed lignocellulose-degrading enzyme activities during the late fermentation stage. Microbial richness declined significantly (Chao index, *p* < 0.05), accompanied by enrichment of thermotolerant taxa such as *Thermobifida* and *Bacillus*, while soluble microbial by-products decreased markedly. Unexpectedly, MGEs including *intI1*, *intI2* and *Tn916/1545* were less abundant under GD exposure, likely reflecting the overall inhibition of microbial growth. These findings demonstrate that GD residues exert cumulative adverse effects on EFS performance, and highlight the need to coordinate on-farm disinfection with downstream manure treatment.

## 1. Introduction

The rapid expansion of intensive husbandry production has significantly increased the global availability of meat, eggs, and dairy products. However, this growth has concurrently triggered a series of environmental challenges, including odorous gas emissions, soil pollution, and river eutrophication. In China, this issue is particularly pressing, with nearly 3.8 billion tons of animal manure generated annually [1]. Data show that when the annual manure output is converted into chemical oxygen demand, total nitrogen (TN), and total phosphorus equivalents, the resulting pollution load has already surpassed that from industrial sources [2]. Therefore, the safe and efficient utilization of livestock waste represents a primary and critical challenge for promoting the sustainable development of modern animal husbandry.

The Ectopic Fermentation System (EFS) is an innovative livestock waste treatment technology derived from the deep-litter system. It is characterized by its ability to simultaneously process both solid animal manure and wastewater via continuous high-temperature aerobic fermentation, with the end fermented product being a semi-finished organic fertilizer [3,4]. Previous research on EFS has primarily focused on enhancing treatment effectiveness through strategies such as inoculating with functional microbial agents or modifying fermentation processes [3,4,5]. These studies have demonstrated the excellent performance of EFS in treating livestock waste, and this technology has been widely applied in intensive livestock farms [6].

As is well-established, intensive livestock and poultry operations normally employ standardized disinfection measures to eliminate pathogenic microorganisms in the environment, thereby mitigating the risk of animal disease outbreaks [7,8]. Farm managers typically select disinfectants based on factors such as cost, bactericidal efficacy, and operational requirements. Among the available options, glutaraldehyde-didecyldimethylammonium bromide (GD) is widely adopted for environmental disinfection in livestock and poultry housing due to its high efficacy and cost-effectiveness [7]. While these standardized disinfection procedures are crucial for disease prevention, residual disinfectants from the environment can enter the farm’s manure storage system via pipelines and subsequently interfere with the post-treatment of livestock waste. According to the product guidelines, the typically effective concentration of GD ranges from 12.5 to 100 mg/L, though in practice, its concentrations may significantly exceed this range. It has been reported that even 0.002%~0.08% glutaraldehyde can significantly inhibit methane production in anaerobic fermentation systems treating livestock wastewater, reduce nitrogen and carbon removal efficiency [9], and 10 mg/L GD can elevate the risk of horizontal and vertical transfer of antibiotic resistance genes (ARGs) [7]. In addition, when animal manure containing residual quaternary ammonium disinfectants (e.g., benzalkonium chloride) is applied as fertilizer, it can increase the risk of spreading drug-resistant pathogens in soil environments [10]. The mechanisms by which residual disinfectants may influence ARG dissemination include the enhancement of quorum sensing [11], flagellar motility, and cell membrane permeability [12]. Therefore, the impact of residual disinfectants on the aerobic fermentation process, particularly under conditions of continuous manure slurry loading (e.g., EFS), remains unclear and needs further investigation.

Given the microbicidal effect of disinfectants, this study hypothesizes that low-dose GD may influence the continuous fermentation performance of EFS by inhibiting microbial activity. Therefore, this study aimed to elucidate the impact of residual GD in pig manure slurry on EFS performance by integrating multiple physicochemical parameters, 3D excitation–emission matrix (3D-EEM) fluorescence analysis, metagenomics, and quantitative analysis of ARGs and mobile genetic elements (MGEs). The findings provide theoretical guidance for the stable operation of EFS and the safe utilization of its end-fermentation product.

## 2. Materials and Methods

### 2.1. Preparation of the Ectopic Fermentation System and Experimental Design

Rice husks and corn stalks were mixed in a 1:4 ratio to serve as the EFS bedding materials. All bedding materials were purchased from the Taobao platform and were confirmed to be mold-free. Afterwards, the EFS bedding materials were thoroughly mixed with pig manure slurry (collected from Chengdu Yuanwei Tianxiang Agricultural Co., Ltd., Chengdu, China), and moisture content was adjusted to ~50%. Finally, the mixture was transferred to six separate fermentation vessels (~37.5 L each) for spontaneous fermentation. Glutaraldehyde (50%, *w*/*w*, aqueous solution, analytical reagent grade) and didecyldimethylammonium bromide (DDAB, 80%, *w*/*w*) were used as the model disinfectants. A stock solution was freshly prepared before each loading by dissolving 4.00 g of the glutaraldehyde solution (equivalent to 2.00 g glutaraldehyde) and 2.50 g of the DDAB product (equivalent to 2.00 g DDAB) in double-distilled water (ddH_2_O) to a final volume of 100 mL, giving 20 mg/kg of each active ingredient (1:1, *w*/*w*). For each loading, 1.00 mL of the stock solution was added to 998 g of fresh slurry, corresponding to a nominal concentration of 20 mg/kg of each compound (40 mg/kg total disinfectant) on a wet-weight slurry basis. The slurry used to establish the fermentation bed was dosed at the same rate.

The detailed experimental design and sampling methods are shown in Table 1.

### 2.2. Determination of Physicochemical Parameters

The EFS fermentation was conducted for a total of 24 days. All samples were collected immediately before each slurry loading. Fermentation and ambient temperatures were recorded daily using an electronic thermometer. Fresh fermentation samples were dried in an oven at 105 °C for 24 h. After equilibrating to constant weight, the moisture content (MC) was calculated based on the sample weight loss. The pH value (Mettler Toledo FE28-standard, Greifensee, Switzerland) was measured after extracting fresh samples with CO_2_-free ddH_2_O at a weight-to-volume ratio of 1:10 (*w*/*v*). The air-dried samples were pulverized for the determination of other physicochemical parameters. Specifically, electrical conductivity (EC, Qiwei DDS-11A, Hangzhou, China) was measured by the previously described method [13]. TN and total organic carbon (TOC) contents were determined by Kjeldahl digestion and K_2_Cr_2_O_7_ oxidation methods, respectively. For the determination of soluble carbohydrates, freeze-dried fermentation samples were extracted with ddH_2_O (*w*/*v* = 1:20). The extract was filtered through a 0.45 μm filter and analyzed using the anthrone method [3].

### 2.3. Germination Index (GI)

Air-dried fermentation samples were extracted with ddH_2_O (*w*/*v* = 1:10). Subsequently, 10 evenly sized and plump *Brassica rapa* subsp. *chinensis* seeds (Qingxian Chunfeng Seed Industry Co., Ltd., Cangzhou, China) were selected and evenly distributed on a 9 cm dish lined with filter paper. Then, 8 mL of extract solution was added to each dish (the control group received ddH_2_O), and the dishes were incubated in the dark at 25 °C. Each sample was tested in triplicate. After 48 h of incubation, the number of germinated *Brassica rapa* subsp. *chinensis* seeds was counted, and root length was measured in each dish.

Germination was determined according to the following criteria: (a) Seed coat rupture is considered germination. (b) Seed coat ruptured but no sprout emerged (root length = 0 mm). The seed germinates, with a long shoot but no root hairs (root length = 1 mm). The seed germinates and grows root hairs; root length is measured starting from the root hair (root length > 1 mm).

The equation for calculating GI is as follows [14]:

$$\mathrm{GI}=\frac{\mathrm{Number}\;\mathrm{of}\;\mathrm{gerninated}\;\mathrm{seeds}\;\mathrm{in}\;\mathrm{extract}}{\mathrm{Number}\;\mathrm{of}\;\mathrm{gerninated}\;\mathrm{seeds}\;\mathrm{in}\;\mathrm{control}}\times \frac{\mathrm{Root}\;\mathrm{length}\;\mathrm{in}\;\mathrm{extract}}{\mathrm{Root}\;\mathrm{length}\;\mathrm{in}\;\mathrm{control}}\times 100\%$$

### 2.4. Analysis of Lignocellulolytic Enzyme Activity

Lyophilized EFS fermentation sample (1.000 g) was accurately weighed into a 50 mL sterile centrifuge tube. Then, 1 mL toluene was added, and the mixture was allowed to stand for 15 min; extraction buffer of crude enzyme was added (*w*:*v* = 1:40, Supplementary Materials). Finally, all tubes were shaken at 25 °C and 220 rpm for 1 h to obtain the crude-enzyme liquid. The resulting crude-enzyme liquid was used to determine the activities of laccase (ABTS method), β-glucosidase (p-Nitrophenol colorimetric method), and cellulase (DNS method), respectively [15].

### 2.5. Metagenomic Library Preparation and Analysis

Total genomic DNA was extracted from 30 freeze-dried EFS samples (500 mg per sample) using the CTAB method [3]. The quality of DNA was determined by SynergyHTX (Agilent BioTek, Winooski, VT, USA), NanoDrop2000 (Thermo Fisher Scientific, Wilmington, DE, USA), respectively, and DNA integrity was checked on a 1.2% agarose gel. Subsequently, the total genomic DNA was fragmented to an average size of ~350 bp using M220 Focused-ultrasonicator (Covaris, Woburn, MA, USA) for paired-end library construction. A paired-end library was constructed using the NEXTFLEX Rapid DNA-Seq Kit (Bioo Scientific, Austin, TX, USA). Paired-end sequencing was performed on the Illumina NovaSeq^TM^ X Plus platform (Illumina, San Diego, CA, USA) at Majorbio Bio-Pharm Technology Co., Ltd. (Majorbio, Shanghai, China).

Specifically, shotgun metagenome raw reads were quality-filtered using Fastp (v0.20.0) to remove low-quality reads and adapter sequences [16]. Then, MEGAHIT (v1.2.9) was used for cleaned data assembly [17]. After this, open reading frames (ORFs) were predicted from assembled contigs using Prodigal (v2.6.3) [18], and the non-redundant gene catalog of each sample was clustered using CD-HIT (v4.6.1, 95% identity and 90% coverage thresholds) [19]. Subsequently, this non-redundant gene set was aligned against the NCBI NR database (20230830 release) using Diamond v2.0.13 [20]. Gene abundance was estimated by mapping clean reads back to the non-redundant gene set using SOAPaligner (soap2.21 release). For diversity analyses, reads were rarefied to 10,000,000 reads per sample before calculating Chao and Shannon indices.

### 2.6. 3D Excitation-Emission Matrix Spectra

The dissolved organic matter (DOM) was extracted from the lyophilized EFS sample using ddH_2_O (*w*:*v* = 1:20) and continuously shaken on an oscillator at 200 rpm, 25 °C for 24 h. Then, the EFS sample was centrifuged at 12,000 g, and the supernatant was filtered through a 0.45 μm membrane filter. An 8 mL filtrate was accurately transferred into a glass tube and dried at low temperature (50 °C). The dissolved organic carbon (DOC) of the filtrate liquid was determined using the K_2_Cr_2_O_7_ oxidation method. Afterwards, the DOC concentration in each sample was adjusted to 10 mg/L using ddH_2_O.

The 3D-EEM spectra were obtained using the fluorescence spectrophotometer (Hitachi FP-7000, Tokyo, Japan). The excitation wavelength (Ex) was from 200 to 500 nm with 8 nm increments, and the emission wavelength (Em) was from 200 to 600 nm with 1 nm increments (scanning speed of 2400 nm/min). The 3D-EEM spectral data were corrected and normalized using TreatEEM software. Then, we used the fluorescence regional integration (FRI) method described by Chen et al. [21] to further quantify the spectral regions of 3D-EEM spectra.

### 2.7. Absolute Quantification qPCR

The target ARGs were amplified using the primer pairs listed in Table S1, and fragments of target genes were purified using the DNA Gel Extraction Kit (Tsingke TSP502-200, Beijing, China). The purified DNA fragments were ligated into the TOPO vector via TA cloning to generate standard plasmids for absolute quantification PCR, following the operating manual of the Ultra-Universal TOPO Cloning Kit (Vazyme C603-01, Nanjing, China). Then, the positive plasmid was extracted and the plasmid concentration was diluted to 10^1^~10^9^ copies/μL. The qPCR reaction was performed on the LightCycler^®^ 96 platform (Roche, Basel, Switzerland). The primer pairs and qPCR amplification protocols used in this study are provided in the Supplementary Materials. Finally, the amplification efficiency of all ARGs was limited to between 90% and 110%, and the relative abundance of each ARG was calculated as target ARG copies/16S rRNA copies.

### 2.8. Statistical Analysis

The physicochemical parameters, enzyme activity, and ARG abundances were analyzed using SPSS (v19.0) and GraphPad Prism (v8.0). Metagenomic data were visualized and analyzed using the online Majorbio Cloud Platform (www.majorbio.com). The variance partition analysis (VPA) was performed using the vegan package (v2.6.4) in R (v4.2.0). Statistical significance was determined by ANOVA followed by Tukey’s test or by Student’s *t*-test; ‘ns’ represents no significance, *p* > 0.05, ‘*’ represents *p* < 0.05, ‘**’ represents *p* < 0.01, ‘***’ represents *p* < 0.001.

## 3. Results and Discussion

### 3.1. Effects of GD on Physicochemical Parameters and Germination Index

Temperature is a critical indicator reflecting the performance of EFS during continuous fermentation. As shown in Figure 1a, the fermentation temperature in both the CK and GD groups gradually increased, reaching 60 °C on day 4. Thereafter, the fermentation temperature exhibited periodic fluctuations with the supplementation of pig manure slurry. Notably, the supplementation of pig manure slurry containing GD significantly enhanced the fermentation temperature on days 8, 16, 20, 21, 22, and 24, compared to the CK group (*p* < 0.05). It is plausible that under spontaneous fermentation conditions, the presence of GD may exert selective pressure on the microbial community [22], potentially enriching thermotolerant populations and thereby increasing fermentation temperature. This hypothesis is supported by recent findings showing that glutaraldehyde can selectively enrich stress-tolerant microorganisms such as *Firmicutes* in pig manure fermentation systems [9]. Additionally, glutaraldehyde-didecyldimethylammonium bromide (DAB) has been demonstrated to alter microbial community composition by selectively suppressing certain taxa while promoting the proliferation of resistant populations [23]. The enrichment of these stress-tolerant and potentially thermotolerant microorganisms may contribute to the elevated fermentation temperatures observed in the GD group.

Moisture content and pH are important for the stable and efficient operation of EFS (Figure 1b,c). Previous studies have recommended that moisture content should be maintained between 40 and 70% to provide a suitable environment for microbial fermentation [5,24]. In this study, moisture content gradually increased over time and stabilized within the range of 60 to 70% from days 8 to 24. Notably, moisture content in the CK group was consistently higher than that in the GD group between days 8 and 24, with a statistically significant difference observed on day 16 (*p* < 0.01). This suggests that the higher fermentation temperatures observed in the GD group may have promoted moisture evaporation. For pH, a significant difference between groups was observed only on day 0 (*p* < 0.05). As fermentation continued, pH in both CK and GD groups initially decreased from day 0 to 2 and then increased from day 8 to 24, ultimately maintaining around 9.0. Unlike the continuous increase or an initial increase followed by a decrease in EC typically observed in traditional composting processes [25,26], EFS exhibited a trend of first decreasing and then increasing for EC value (Figure 1d). This pattern is probably attributed to the increase in ion concentration resulting from the sustained supplementation of pig manure slurry in the EFS.

As shown in Figure 1e,f, the effects of pig manure slurry containing 20 mg/kg GD on TN and TOC of EFS fermentation samples were investigated. The results showed that TN content in the CK group increased from 8.00 ± 0.69 mg/g to 21.61 ± 0.66 mg/g, while TN content in the GD group increased from 8.50 ± 0.62 mg/g to 20.32 ± 0.27 mg/g. At the end of fermentation (24 d), the TN content in the CK group was significantly higher than that in the GD group (*p* < 0.05), suggesting that 20 mg/kg GD in pig manure slurry affected nitrogen retention during fermentation, as both groups received an identical nitrogen input. This difference is likely related to the balance of nitrogen transformation pathways in EFS. Proteinaceous nitrogen is first mineralized to NH_4_^+^/NH_3_ by ammonification. The strongly alkaline (Figure 1c) and thermophilic (Figure 1a) conditions favor free ammonia formation and NH_3_ volatilization, whereas autotrophic nitrification is expected to be largely inhibited at such temperatures and free-ammonia levels. Denitrification may still proceed in the anaerobic microsites of the moist bedding matrix. The higher temperature and lower moisture content of the GD group (Figure 1a,b) would therefore be expected to intensify NH_3_ loss. As gaseous emissions and NH_4_^+^-N/NO_3_^−^-N pools were not monitored here, these pathways are inferred rather than directly quantified. However, 20 mg/kg GD addition did not affect the degradation of TOC in the fermented samples. By the end of fermentation, the TOC content in both groups remained between 413.00 and 414.82 mg/g. In this study, GD enhanced the fermentation temperature of EFS to a certain extent (Figure 1a). Our previous work indicates that the soluble carbohydrate content in EFS can effectively enhance fermentation temperature [3]. Thus, we further assayed the levels of soluble carbohydrates in all samples (Figure 1g) and found that they were significantly higher in the GD-treated group on day 8 and 16 compared to the CK group (*p* < 0.05). Specifically, in the GD group, soluble carbohydrate levels were 49.39 ± 7.07, 41.92 ± 14.59, 9.67 ± 0.13, 7.66 ± 0.14, and 2.87 ± 0.17 mg/g on days 0, 2, 8, 16, and 24, respectively.

The germination index is an important indicator reflecting the maturity and phytotoxicity of compost [27]. As a semi-finished fertilizer, EFS fermentation products can also be evaluated using the GI method described above [28]. Here, we evaluated the toxicity of fermentation products to *Brassica rapa* subsp. *chinensis* seeds during the entire fermentation process. The results showed that GI in both the CK and GD groups increased as fermentation progressed (Figure 1h). The presence of GD in pig manure slurry reduced GI on days 2, 8, 16, and 24. Among these, GI in the GD group on days 8 and 16 was significantly lower than that in the CK group (*p* < 0.05). These results indicate that residual GD in the pig manure slurry aggravated the phytotoxicity of the fermented material, whereas the difference between the two groups diminished by the end of fermentation, suggesting that this adverse effect was gradually alleviated as fermentation proceeded.

### 3.2. Analysis of Lignocellulose-Degrading Enzyme Activity in EFS

Previous studies have demonstrated that the activity of lignocellulose-degrading enzymes is fundamental to sustaining the EFS fermentation process [3,29]. To elucidate the impact of 20 mg/kg GD-treated pig manure slurry on these enzymatic apparent activities, we monitored the dynamic pattern of laccase, β-glucosidase, and cellulase apparent activities in the bedding materials throughout the fermentation. Laccase apparent activity improved primarily during the later period of fermentation (Figure 2a), consistent with the findings reported by a previous study [3]. Notably, although 20 mg/kg GD reduced laccase apparent activity throughout the entire EFS fermentation period, the inhibition did not reach a statistically significant level (*p* > 0.05). In contrast, on day 8, 20 mg/kg GD significantly enhanced the β-glucosidase and cellulase apparent activities in the bedding materials (Figure 2b,c, *p* < 0.05). However, with prolonged fermentation and continuous supplementation of pig manure slurry (containing 20 mg/kg GD), β-glucosidase and cellulase apparent activities in the GD group were lower than those in the CK group. These results suggest that short-term exposure to low-dose disinfectants may transiently stimulate microbial metabolic activity, as reflected by the enhanced lignocellulolytic enzyme activities. This transient stimulation may represent an adaptive response of the microbial community to low-level chemical stress, whereby microorganisms temporarily upregulate metabolic activities to counteract environmental perturbations. Such biphasic responses to antimicrobial agents are commonly interpreted as hormesis [30], and a comparable pattern has been reported for GD specifically, with sub-inhibitory concentrations activating oxidative-stress responses and enhancing energy metabolism in activated sludge, whereas higher concentrations suppressed enzyme activities [7]. However, with prolonged exposure, the inhibitory effects of disinfectants on microbial metabolism became apparent, as evidenced by the reduced enzyme activities in the GD group. It should be noted that intracellular ROS, ATP, and stress-related gene expression were not measured in this study, and this mechanism therefore remains an interpretation requiring further verification.

### 3.3. Effects of GD on the Microbial Composition During EFS Fermentation Process

In this study, 30 fermentation samples were collected on days 0, 2, 8, 16, and 24 for metagenomic sequencing. During EFS fermentation, the α-diversity index gradually increased and stabilized (Figure S1). In particular, the GD group exhibited significantly lower Chao indices than the CK group on day 8 (*p* < 0.05) and day 24 (*p* < 0.01), suggesting that GD may have reduced microbial species richness during these periods by exerting selective pressure on the microbial community. The Shannon index showed the same directional pattern, being numerically lower in the GD group on days 8, 16, and 24, although the differences were not statistically significant (*p* > 0.05, Figure S1b).

Regarding microbial composition, Figure 3a presents the relative abundance of samples at the genus level (Top 20), respectively. At the initial stage of fermentation (day 0), the dominant microorganisms primarily consisted of *Prevotella*, *unclassified_c__Caudoviricetes*, *Lactobacillus*, *Clostridium*, *Streptococcus*, *Treponema*, *Bacteroides*, *Limosilactobacillus*, *Oscillibacter*, and *Segatella*. As fermentation progressed, the microbial community structure of the EFS bedding materials changed dramatically, resulting in *Pseudoxanthomonas*, *Luteimonas*, *unclassified_p__Candidatus Cloacimonadota*, and *Thermobifida* becoming the dominant genera. Notably, on days 16 and 24, the Top 20 abundance of microbial genera in the GD group reached 39.99% and 38.67%, respectively, both higher than those in the CK group. These results indicate that a low concentration of GD apparently influences the fermentation process by altering the microbial composition of the EFS bedding materials.

Figure 3b presents the clustering heatmap of genus-level abundance across different fermentation periods,. The microbial communities in the bedding material are roughly divided into four stages: the initial fermentation phase (0 d), the heating phase (2 d), the high-temperature phase (8 d), and the fermentation stabilization phase (16 d and 24 d). This clustering pattern is consistent with the PCoA analysis (Figure 3c). Although GD in pig manure slurry did not alter the number of core species throughout the fermentation period (Figure S2), it increased the abundance of thermotolerant microorganisms (Figure 3b), such as *Thermobifida*, *Caldibacillus*, and *Bacillus*, possibly due to their greater resistance to disinfectants. These thermotolerant bacteria are known to possess robust lignocellulolytic enzyme systems adapted to extreme conditions [31,32], which may account for the transient enhancement of β-glucosidase and cellulase activities during 8d (Figure 2b,c), and the higher fermentation temperature in the GD group (Figure 1a). Importantly, on day 24, GD led to a significant reduction in *Pseudomonas* abundance. *Pseudomonas* is known to regulate nitrogen cycling during composting as a functional bacterium, thereby contributing to improved compost product quality [33]. Therefore, the reduction in *Pseudomonas* abundance may represent a critical factor contributing to the significantly lower TN content in the GD group compared to the CK group on day 24 (*p* < 0.05, Figure 1e). However, it should be noted that this association is based on correlational evidence in this study, and direct measurements of nitrogen-cycling functional genes (such as *nifH*, *nirK*, and *nosZ*) would be necessary to confirm the mechanistic link between the reduction in *Pseudomonas* and the observed difference in TN concentration. Such measurements have proved informative in comparable systems, in which *Pseudomonas* spp. were identified as core nosZ-harboring denitrifiers during manure composting and qPCR profiling revealed the effects of a chemical additive on nitrogen conversion in pig manure compost [34]. The association proposed here should thus be regarded as a working hypothesis.

### 3.4. Effects of GD on 3D-EEM-Derived Profiles

Previous studies have employed the 3D-EEM method to characterize dissolved organic matter (DOM) in compost and EFS extracts [3,35,36]. Here, to further elucidate the effects of 20 mg/kg GD treatment on DOM composition, we performed 3D-EEM analysis on samples collected at different fermentation stages and integrated the fluorescence areas across I to V regions using the FRI method. As previously described, regions I to V correspond to tyrosine substances (Ex/Em, 200~250 nm/200~330 nm), tryptophan (Ex/Em, 200~250 nm/330~380 nm), fulvic acids (Ex/Em, 200~250 nm/380~600 nm), soluble microbial by-products (Ex/Em, 250~500 nm/200~380 nm), and humic acid-like substances (Ex/Em, 250~500 nm/380~600 nm), respectively [3].

As ectopic fermentation progressed, the 3D-EEM spectral intensity in region V changed markedly in both the CK and GD groups (Figure 4a), suggesting that the humification degree and maturation of the EFS bedding materials progressively increased. Figure 4b presents the FRI results for different regions of the 3D-EEM spectra. With the exception of Region I, the absolute fluorescence intensity integration values for regions II to V increased with fermentation time, whereas the percentage changes for these regions exhibited no discernible pattern (Figure S3). Notably, on fermentation days 8, 16, and 24, both the absolute (Figure 4b) and percentage (Figure S3) fluorescence intensity values of region IV in the GD group were lower than those in the CK group. Previous studies have indicated that higher levels of soluble microbial by-products may be more favorable for composting progression [7]. While 20 mg/kg GD in manure slurry may initially promote thermophiles (e.g., *Thermobifida*) and temporarily increase temperature (Figure 1a), its continuous supplementation likely inhibits microbial activity, reduces soluble microbial by-products (Figure 4b), decreases lignocellulolytic enzyme activity during the later stage of fermentation (Figure 2), and may ultimately lead to impaired maturity.

### 3.5. Effects of GD on ARGs

Considering that low doses of disinfectants can act as non-antibiotic factors promoting the transfer and spread of ARGs in the environment [7,37], we thereby employed absolute qPCR technology to analyze the abundance of the 16S rDNA gene and ARGs in the samples. On day 0, the absolute abundances of the 16S rDNA gene in the CK and GD groups were 1.73 × 10^12^ and 1.31 × 10^12^ copies g^−1^, respectively. As fermentation progressed, the abundance of the 16S rDNA gene exhibited a trend of first decreasing, then increasing, and finally decreasing again. Importantly, samples treated with 20 mg/kg GD resulted in reduced absolute abundance of the 16S rDNA gene throughout the entire fermentation process, suggesting that the low dose of disinfectant contained in the manure slurry may continuously reduce microbial growth (Figure 5a). Figure 5b,c show the absolute and relative abundances of bacterial mobile genetic elements (MGEs), respectively. Unexpectedly, samples containing GD-treated manure show a reduction in the absolute abundance of MGEs during days 2 to 24 and the relative abundance of MGEs during days 2 to 16. On day 24, the absolute abundances of *intI1*, *intI2*, *Tn916/1545*, and *IncQ oriT* in the CK group were 6.17 × 10^9^, 1.88 × 10^9^, 1.23 × 10^9^, and 2.54 × 10^9^, respectively. The corresponding values in the GD group were 1.87 × 10^9^, 5.05 × 10^8^, 4.76 × 10^8^, and 1.15 × 10^9^, respectively. Consistent with this study, evidence from in vitro and pig manure composting experiments suggests that the proper use of disinfectants does not increase antibiotic resistance in *E. coli*, and that ozone can even inhibit the horizontal transfer of ARGs [38,39]. Figure 5d,e further illustrate the profiles of ARG and MGEs abundances. Notably, ARGs and MGEs content continued to increase throughout fermentation, which is potentially attributed to the active microbial community resulting from the continuous decomposition of organic matter in the EFS. At the genus level, several ARGs co-varied positively with *Pseudomonas* and *Halopseudomonas* (Figure S4). It should be noted, however, that such co-occurrence patterns cannot be taken as evidence of host identity. Positive correlations may also arise from parallel responses of unrelated populations to shared environmental drivers, such as the pronounced temporal changes in temperature, moisture and available carbon during fermentation, and many ARGs are carried on plasmids or integrons whose abundance is not tied to any single lineage—a possibility supported here by the high abundance of *intI1*, *intI2*, *Tn916*/*1545* and *IncQ oriT*. The taxa identified in Figure S4 are therefore regarded only as candidate taxa for hypothesis generation, and no host assignment is claimed. Unambiguous identification of ARG hosts and their fate under disinfectant stress requires genome-resolved approaches, such as metagenomic binning combined with long-read sequencing, which represents a limitation of the present study and an important direction for future work.

The observation that GD reduced ARG and MGE abundances contrasts with previous studies reporting enhanced antimicrobial resistance dissemination under disinfectant stress. Several non-mutually-exclusive hypotheses, none of which was directly tested here, may account for this discrepancy: (1) In early fermentation, ARGs-carrying bacteria in the manure slurry treated with 20 mg/kg GD may have been partially inactivated or eliminated before horizontal gene transfer could effectively occur. (2) The continuous reduction in overall microbial biomass under GD exposure (Figure 5a) may have limited opportunities for ARG transfer. (3) The selective enrichment of thermotolerant genera (*Thermobifida*, *Caldibacillus*) that are not typically associated with ARGs may have diluted the relative proportion of resistance genes within the community, in line with the close coupling between ARG dynamics and host taxa composition during composting [40]. Discriminating among these possibilities would require quantification of mobile genetic elements (e.g., intI1) together with ARG host tracking, which was beyond the scope of this study.

### 3.6. Redundancy, Variance Partitioning and Correlation Analyses

To further examine how the physicochemical properties of the fermented material co-varied with the lignocellulolytic enzyme activities and the microbial community during EFS, RDA was performed (Figure 6). The first two axes explained 73.06% and 14.39% of the variation, respectively (87.45% in total). In the ordination space, the three enzyme activities (laccase, cellobiase and cellulase) were closely aligned with temperature, moisture content, pH, EC and TN, and oriented oppositely to TOC and soluble carbohydrates, indicating that enzyme activities co-varied positively with the former group of parameters and negatively with the latter. It should be noted that RDA describes co-variation rather than causality, and the directionality of these relationships cannot be resolved from the present data.

The core microbial community structure is critical to the maintenance of EFS fermentation. To assess the extent to which physicochemical indicators (PCI) and lignocellulolytic enzyme activities (LEA) co-varied with shifts in microbial community structure, VPA was performed. PCI and LEA jointly accounted for 75.75–77.81% of the explained variation in the genus-level microbial community (Figure 7a,b). In the GD group, the fraction attributable to LEA alone was higher than in the CK group (30.83% vs. 20.1%). Because variance partitioning yields shares of explained variance without associated uncertainty estimates, this difference was not tested statistically and should be regarded as descriptive. It is also important to note that VPA does not resolve directionality: a larger LEA fraction is equally consistent with community shifts driving changes in enzyme activities as with the reverse. We therefore interpret this result only as an indication that community composition and lignocellulolytic activity were more tightly coupled under GD exposure, without implying that GD acted on the community via lignocellulose degradation.

To further investigate the relationships between the aforementioned factors and the abundance of microbial genera (Top 30), we conducted a correlation heatmap analysis. In the CK group, two key bacterial genera, *Weissella* and *Kurthia*, showed a significant positive correlation with N-NO_3_^−^ content (r > 0.6, *p* < 0.01, Figure 7c). A study on nitrogen retention in garden waste composting indicated that *Kurthia* is a key microorganism in the composting nitrogen cycle, enhancing TN and N-NO*_3_*^−^ contents by reducing the abundance of functional denitrifying bacteria [41]. Additionally, *Weissella* and *Kurthia* exhibit a negative correlation with *Flavobacterium* in this study (Figure S5). One possible interpretation of these co-occurrence patterns is that lactic acid–producing and aerobic heterotrophic taxa such as *Weissella* and *Kurthia* may potentially outcompete *Flavobacterium* under the high-temperature conditions of the fermentation matrix, which might be associated with the higher TN concentration retained in the CK group. Nevertheless, this interpretation remains speculative and warrants functional verification through approaches such as pure culture co-cultivation or transcriptomics.

In the GD group, *Saccharomonospora*, *Caldibacillus*, and *Paenibacillus* showed a strong positive correlation with fermentation temperature (r > 0.7, *p* < 0.01, Figure 7d). Relevant studies indicate that these genera are involved in the degradation of complex organic compounds [32,33,34,35,36,37,38,39,40,41,42,43,44,45]. Specifically, *Saccharomonospora* may be a critical bacterium in the degradation of the chloroacetamide herbicide in soil [43], while *Caldibacillus* plays a pivotal role in lignocellulose degradation and carbohydrate metabolism [45]. Therefore, we propose that the continuous supplementation of pig manure slurry containing 20 mg/kg GD may exert selective pressure on the microbial community in the EFS, indirectly promoting the growth of thermotolerant microorganisms and temporarily enhancing fermentation temperature (Figure 1a). However, we acknowledge that this relationship is correlative and does not establish direct causality. Future mechanistic investigations employing functional gene analysis or controlled inoculation experiments would be needed to validate this hypothesis.

## 4. Conclusions

Disinfectants have markedly improved the efficiency of intensive pig farming, but their residues in manure slurry may impair EFS treatment performance. We found that 20 mg/kg GD-treated pig manure slurry significantly altered both microbial community composition and DOM characteristics during fermentation, and reduced lignocellulolytic enzyme activity in the later stages of fermentation. Continuous GD exposure was associated with reduced TN accumulation, potentially limiting the post-utilization value of EFS products.

Nevertheless, this study has the following limitations: (1) The 24-day fermentation period was designed to capture the start-up and heating phase of the EFS, during which nitrogen transformation and microbial succession are most active. Although this period was sufficient to reveal significant effects on microbial communities and fermentation parameters, it may not fully represent actual EFS operational conditions. In particular, the stability of the observed effects over repeated manure-loading cycles, the cumulative effects of prolonged GD exposure on microbial communities and enzyme activity, and the long-term durability of the bedding material remain to be established. Longer-term trials spanning a complete bedding service cycle therefore warrant further investigation. (2) Our study evaluated only a single GD concentration (20 mg/kg), which limits assessment of dose–response relationships and identification of toxicity thresholds. Future studies should include multiple concentration gradients to establish more comprehensive risk assessment frameworks. (3) The laboratory-scale experimental setup may not fully replicate the environmental heterogeneity and operational variability of commercial EFS facilities. Laboratory-scale tests enable precise control of GD concentration and confounding variables to disentangle the independent biological effects induced by GD residues, whereas field conditions are interfered with by multiple co-existing factors. Factors such as seasonal temperature fluctuations, variable manure composition, and different bedding materials may influence the generalizability of our findings. Therefore, further field-scale trials are required to verify the practical responses under real-world production conditions. (4) While GD is commonly used in intensive farming, other disinfectant types (e.g., chlorine-based, phenolic, or oxidizing agents) may exert different effects on EFS performance.

In summary, while intensive livestock farming focuses on increasing productivity, greater oversight of disinfectant use is needed to promote sustainable development of modern animal husbandry. Future research incorporating multiple disinfectant concentrations, longer fermentation periods, and functional analyses of nitrogen-cycling genes would provide more comprehensive guidance for sustainable livestock farm management.

## Figures and Tables

**Figure 1:**
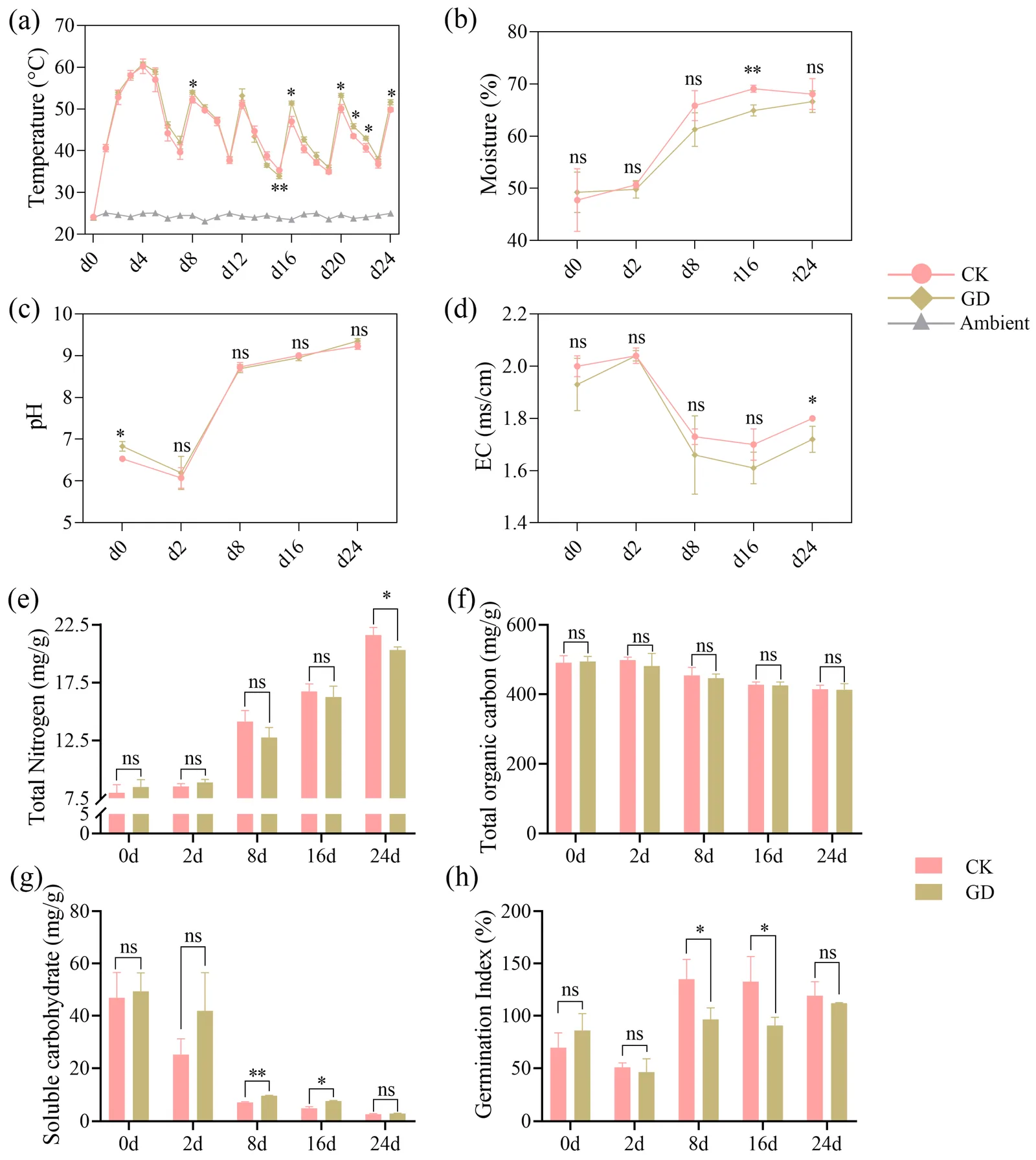
Variation in physicochemical parameters and germination index in the EFS. (**a**) temperature; (**b**) moisture; (**c**) pH; (**d**) electric conductivity; (**e**) total nitrogen; (**f**) total organic carbon; (**g**) soluble carbohydrates; (**h**) germination index. Data are shown as mean ± SD, n = 3 per treatment per time point. ‘ns’ represents no significance, *p* > 0.05, ‘*’ represents *p* < 0.05, ‘**’ represents *p* < 0.01.

**Figure 2:**
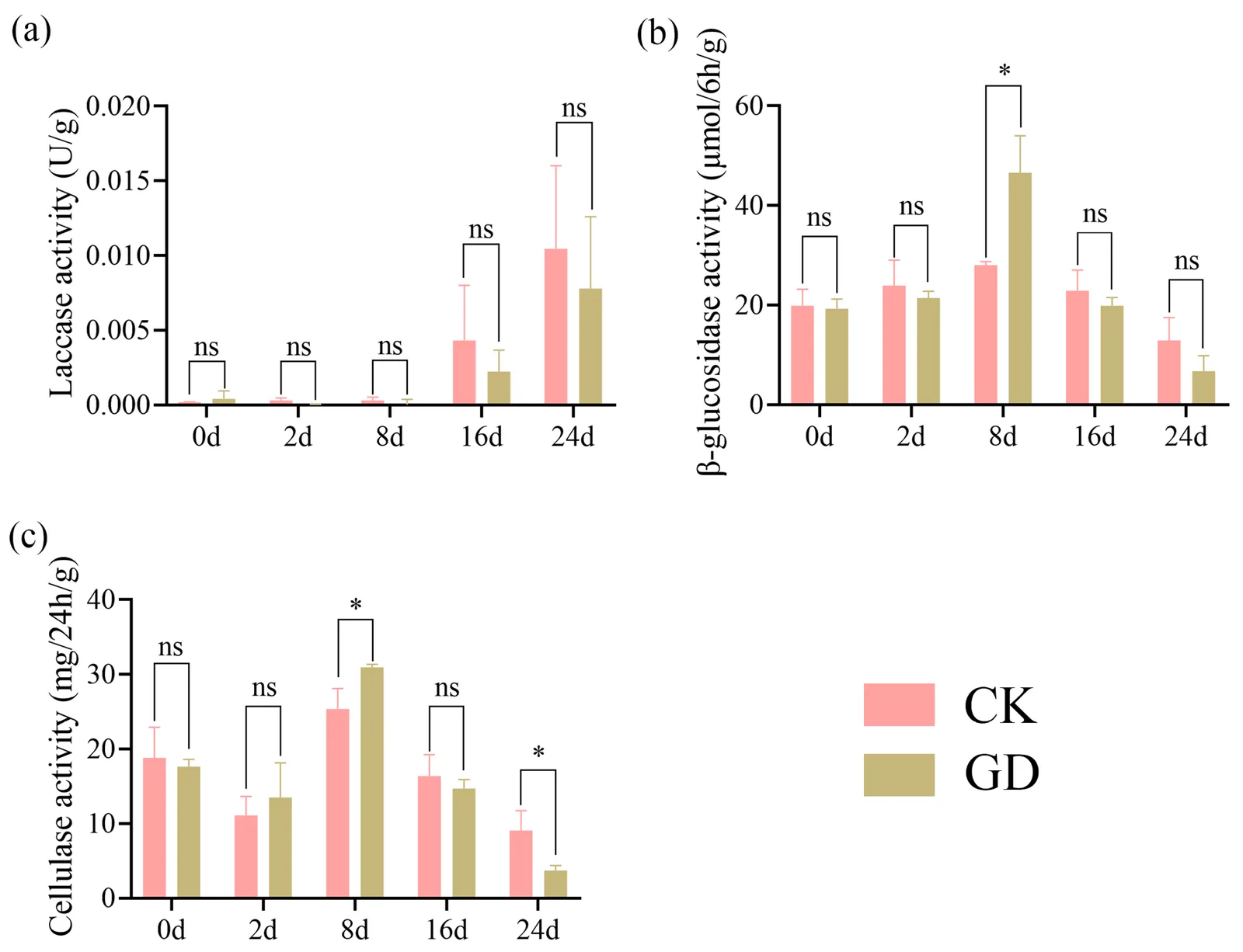
Analysis of lignocellulase activity in the EFS. (**a**) laccase (U/g); (**b**) β-glucosidase (μmol/6h/g); (**c**) cellulase (mg/24h/g). Enzyme apparent activities were measured from crude enzyme extracts, and the extracted buffer is available in the Supplementary Materials. Data are shown as mean ± SD, n = 3 per treatment per time point. ‘ns’ represents no significance, *p* > 0.05, ‘*’ represents *p* < 0.05.

**Figure 3:**
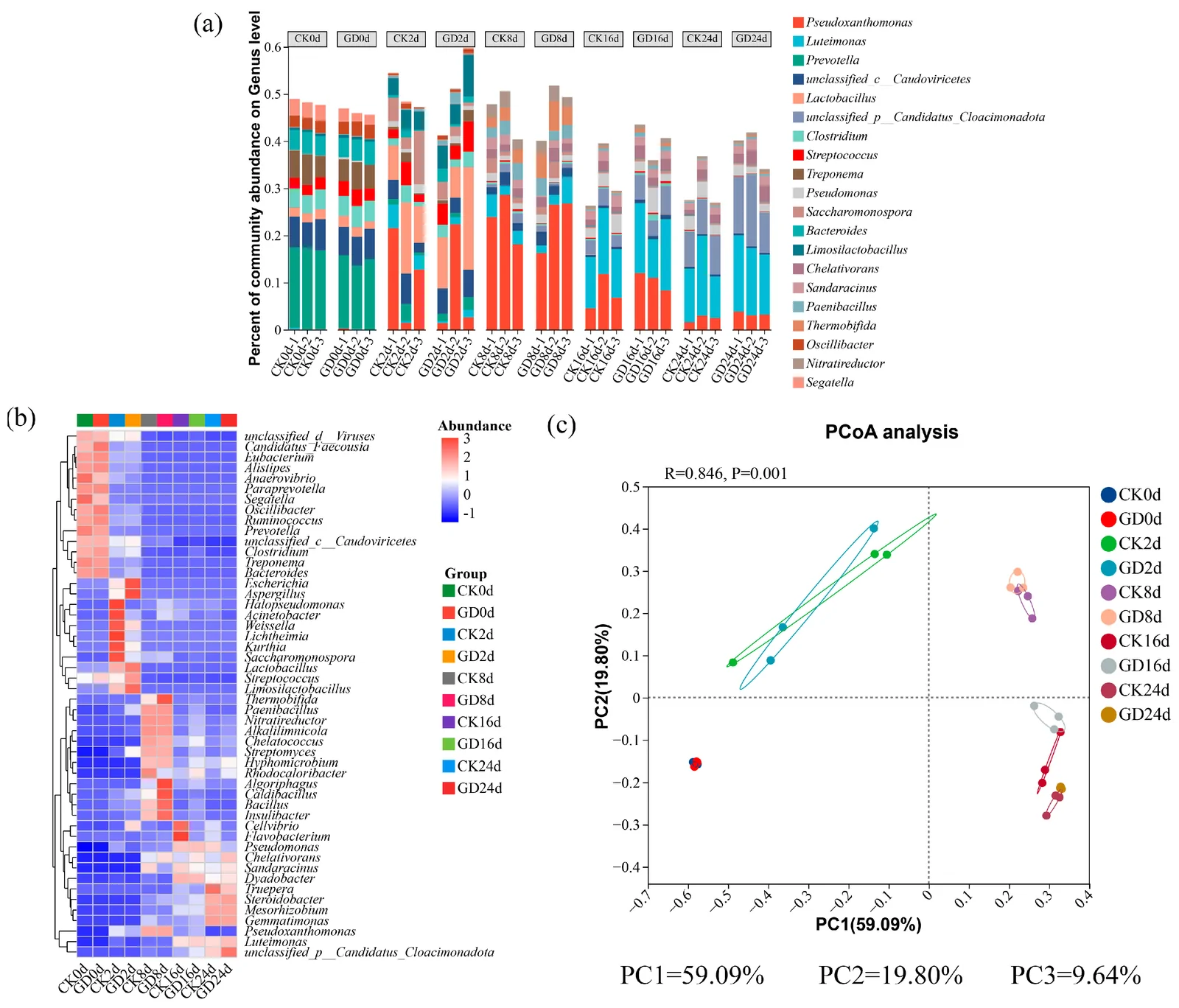
Dynamics of microbial composition and PCoA analysis in the EFS. (**a**) the composition of microorganisms classified by genus level across different samples; (**b**) the heatmap analysis of microbial composition at the genus level across different groups; the heatmap was generated using Bray–Curtis distance and the average linkage (UPGMA) clustering algorithm; (**c**) the PCoA analysis of microbial composition. n = 3 per treatment per time point.

**Figure 4:**
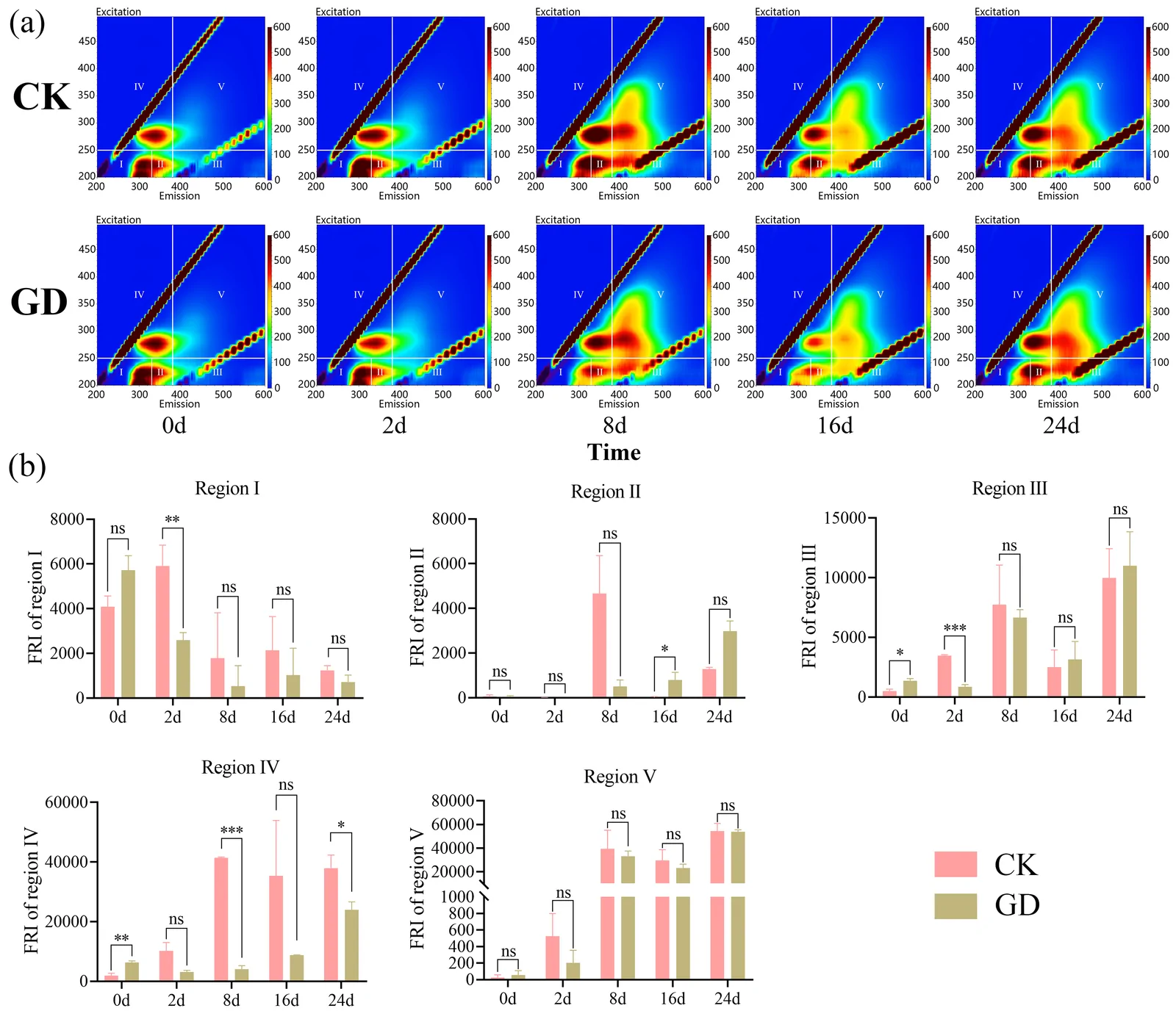
The analysis of 3D-EEM spectra. (**a**) the effect of 20 mg/kg GD on 3D-EEM spectra; (**b**) results of the fluorescence regional integration for I to V regions. Data are shown as mean ± SD, n = 3 per treatment per time point. ‘ns’ represents no significance, *p* > 0.05, ‘*’ represents *p* < 0.05, ‘**’ represents *p* < 0.01, ‘***’ represents *p* < 0.001.

**Figure 5:**
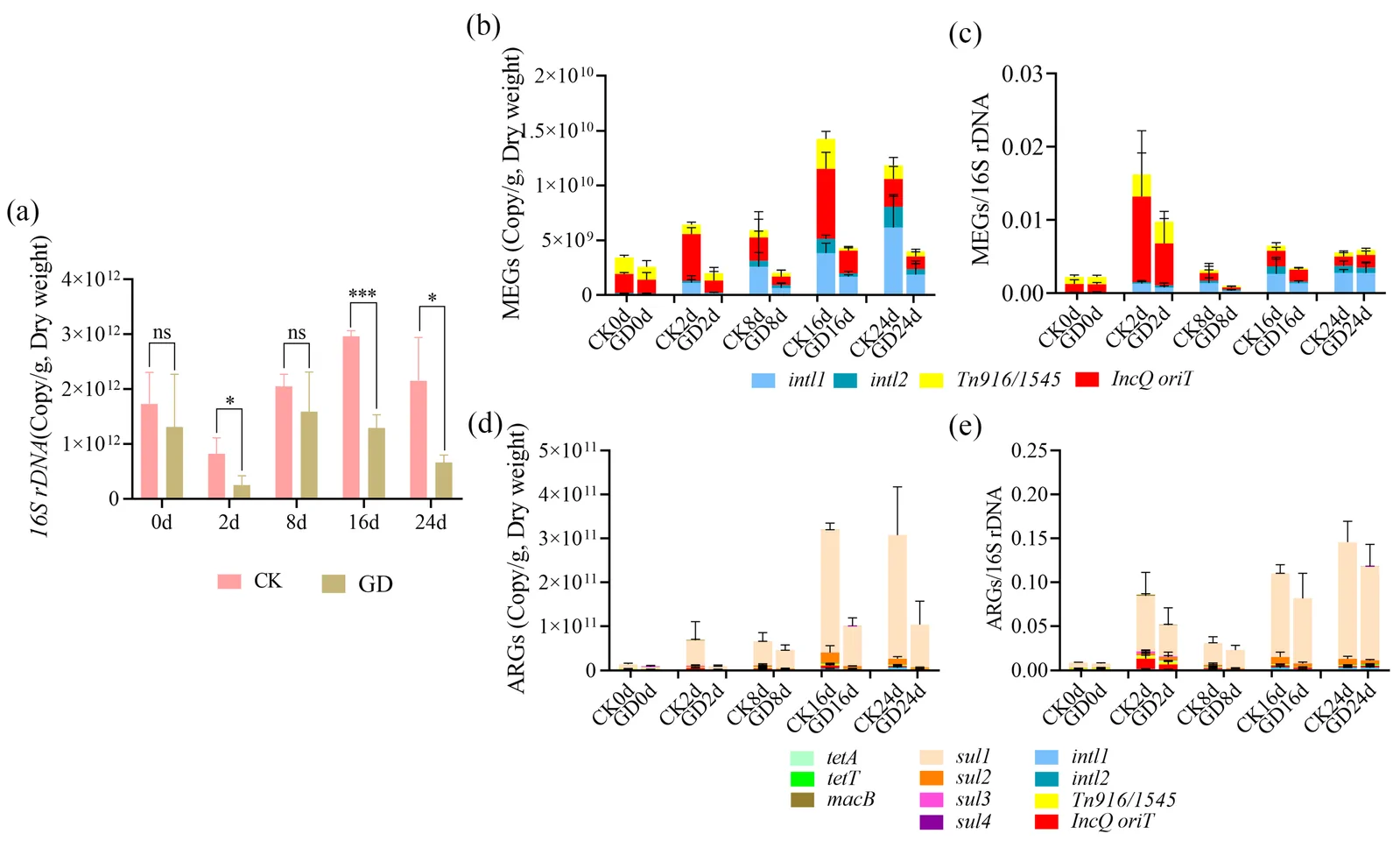
Analysis of antibiotic resistance gene abundance at different fermentation stages in EFS. (**a**) the absolute abundance of 16S rDNA gene; (**b**,**c**) absolute and relative abundances of MGEs, respectively; (**d**,**e**) absolute and relative abundances of both ARGs and MGEs, respectively. Data are shown as mean ± SD, n = 3 per treatment per time point. ‘ns’ represents no significance, *p* > 0.05, ‘*’ represents *p* < 0.05, ‘***’ represents *p* < 0.001.

**Figure 6:**
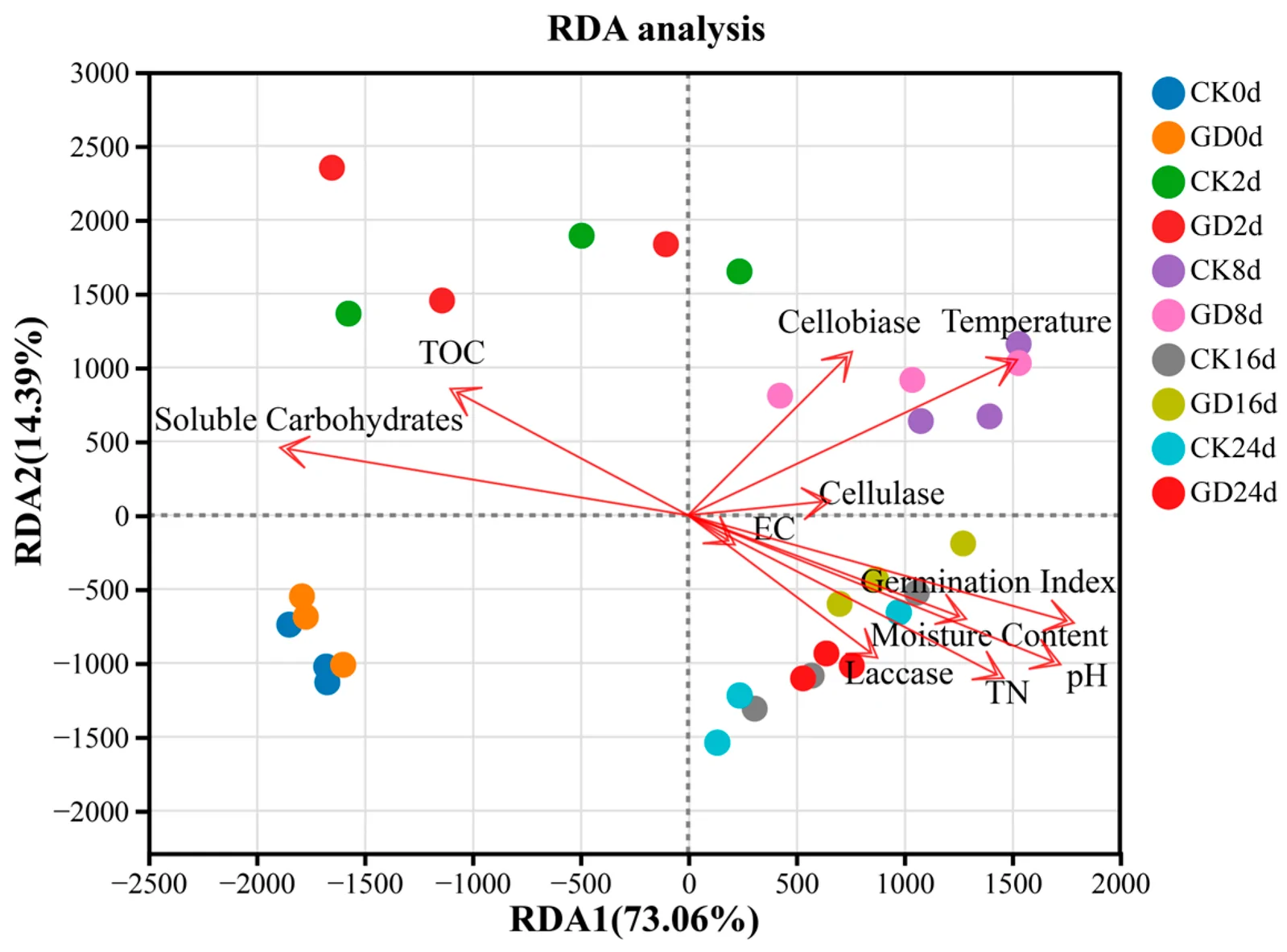
Redundancy analysis (RDA) of the phylum-level microbial community in relation to physicochemical indicators and lignocellulose-degrading enzyme activities during EFS. Explanatory variables include temperature, moisture content, pH, EC, TN, TOC, soluble carbohydrates, germination index, laccase, cellobiase and cellulase.

**Figure 7:**
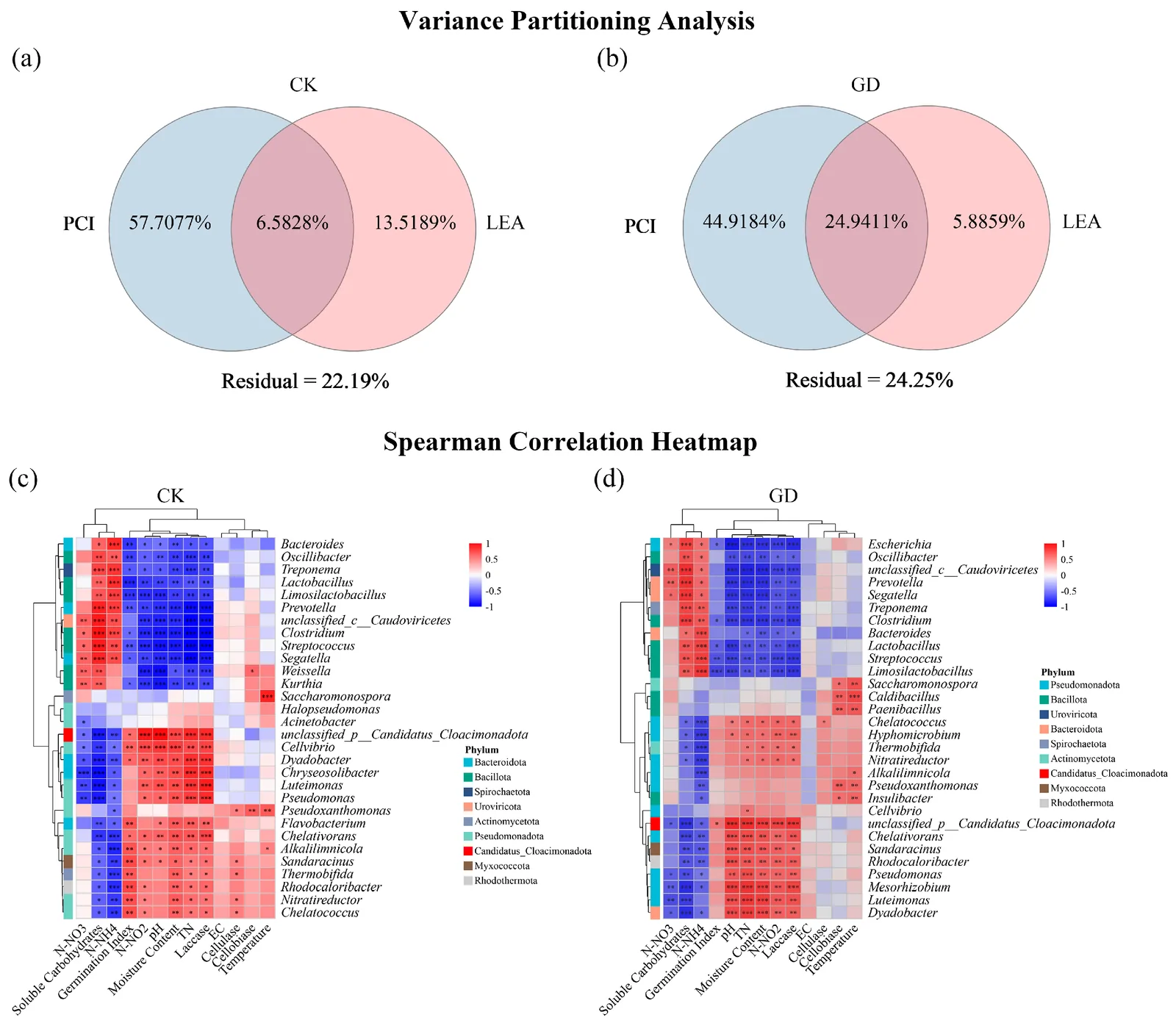
Variance partitioning and Spearman correlation heatmap analyses. (**a**,**b**) VPA of the CK and GD groups, respectively. Physical and chemical indices (PCI) include temperature, moisture content, pH, EC, TN, N-NH_4_^+^, N-NO_2_^−^, and N-NO_3_^−^. Lignocellulose enzyme activity (LEA) includes laccase, β-glucosidase, and cellulase; (**c**,**d**) Spearman correlation heatmap analysis of environmental factors and genus-level microorganisms. ‘*’ represents *p* < 0.05, ‘**’ represents *p* < 0.01, ‘***’ represents *p* < 0.001.

**Table 1:** Experimental Design and Sample Collection.

Items	Treatment Group	
	CK (n = 3)	GD (n = 3)
GD content in pig manure slurry	0 mg/kg	20 mg/kg
bedding materials	Rice husks and corn stalks in a weight ratio of 1:4. Each vessel contains 2.0 kg of bedding materials.	
Pig manure slurry load	Add 1.00 kg of pig manure slurry every 4 days, and mix it thoroughly with the bedding materials.	
Fermentation management	After supplementing with pig manure slurry, fully mix the bedding materials.	
Sample collection	On days 0, 2, 8, 16, and 24, samples were collected from the upper, middle, and lower layers of the bedding materials in each fermentation vessel. After thorough mixing, 200 g of sample was obtained using the quartering method.	
Sample storage	Store at 4, −20, and −80 °C according to experimental requirements.	
Sample crushing	After air-drying, the samples were ground and passed through a 60-mesh sieve for physicochemical parameter analysis.	

Note: Glutaraldehyde (G105905) and didecyldimethylammonium bromide (D399218) were purchased from Shanghai Aladdin Biochemical Technology Co., Ltd., Shanghai, China. The 20 mg/kg concentration refers to the total disinfectant added to the pig manure slurry (glutaraldehyde + didecyldimethylammonium bromide, 1:1 *w*/*w*). To ensure that the pig manure slurry contains no disinfectants, we mix fresh pig manure with ddH_2_O in a 2:3 mass ratio.

## Data Availability

The original contributions presented in this study are included in the article/Supplementary Materials. The raw metagenomic sequencing data in this study have been uploaded to NCBI, BioProject number is PRJNA1492612.

## Supplementary Materials

The following supporting information can be downloaded at: https://www.mdpi.com/article/10.3390/microorganisms14102136/s1. Extraction buffer for enzyme activity analysis; Table S1: The qPCR primer in this study; Table S2: Summary of metagenomic sequencing, assembly, and ORF prediction. Table S3: Individual adjR^2^ of environmental factors for microbial community composition. Figure S1: Dynamics of α-diversity in samples across different time periods; Figure S2: The venn diagrams of shared microorganisms at different time periods.; Figure S3: FRI ratio results in different regions of 3D-EEM; Figure S4: Correlation heatmap of ARGs and microorganisms classified at the genera level (Top 30); Figure S5: The network analysis of microbial correlations at the genus level.

## Abbreviations

The following abbreviations are used in this manuscript:

3D-EEM	3D excitation emission matrix
ARGs	Antibiotic resistance genes
DOM	Dissolved organic matter
EC	Electrical conductivity
EFS	Ectopic fermentation system
FRI	Fluorescence regional integration
GD	Glutaraldehyde-didecyldimethylammonium bromide
GI	Germination Index
LEA	Lignocellulolytic enzyme activities
MC	Moisture content
MGEs	Mobile genetic elements
PCI	Physicochemical indicators
TN	Total nitrogen
TOC	Total organic carbon
VPA	Variance partition analysis
