# Supplementary material for: Impact of Residual Glutaraldehyde-Didecyldimethylammonium Bromide on Fermentation Performance and Microbial Community in Ectopic Fermentation System
Source: Microorganisms. 2026 Sep 22;14(10):2136. doi: 10.3390/microorganisms14102136 (PMC13707770; doi:10.3390/microorganisms14102136)
Supplement: Supplementary file 1 [file microorganisms-14-02136-s001.zip › microorganisms-4527964-supplementary.pdf]

Impact of residual glutaraldehyde-didecyldimethylammonium bromide on fermentation performance and microbial community in ectopic fermentation system

Zhuomacairang Wang [1], Richeng Cui [1], Jiayin Tong [1], Pengliang Liu [1,2,3], Shuntao Huang [4], Yiren Gu [1,2,3,*] and Gan Luo [1,2,3,*]

[1] College of Animal and Veterinary Sciences, Southwest Minzu University, Chengdu 610041, China
[2] Key Laboratory of Qinghai-Tibetan Plateau Animal Genetic Resource Reservation and Utilization, Ministry of Education, Southwest Minzu University, Chengdu, 610041
[3] Key Laboratory for Animal Science of National Ethnic Affairs Commission, Southwest Minzu University, Chengdu 610041, China
[4] Buffalo Research Institute, Chinese Academy of Agricultural Sciences and Guangxi Zhuang Nationality Autonomous Region, Nanning 530001, China
* Correspondence: guyiren1128@163.com (Y.G.); 80300306@swun.edu.cn (G. L.)

Captions

Extraction buffer for enzyme activity analysis.

Table S1. The qPCR primer in this study.

Table S2. Summary of metagenomic sequencing, assembly, and ORF prediction.

Table S3. Individual adjR2 of environmental factors for microbial community composition.

Figure S1. Dynamics of α-diversity in samples across different time periods.

Figure S2. The venn diagrams of shared microorganisms at different time periods.

Figure S3. FRI ratio results in different regions of 3D-EEM.

Figure S4. Correlation heatmap of ARGs and microorganisms classified at the genera level (Top 30).

Figure S5. The network analysis of microbial correlations at the genus level.

Extraction buffer for enzyme activity analysis.

1) Laccase extract buffer: 0.1 M NaAc, 0.005 M CaCl2, 0.05% Tween 80, and 1% polyvinylpolypyrrolidone.

2) β-glucosidase and cellulase extract buffer: 0.1 M phosphate buffer (pH = 7.0), 0.3 M KCl, 10 mM EDTA.

Table S1. The qPCR primer in this study.

Target Gene	Primer Sequences (5′ – 3′)	Amplicon Size (bp)	Annealing Temp (^{o}C)
*tet*A	F：GCTACATCCTGCTTGCCTTC<br>R：CATAGATCGCCGTGAAGAGG	210	55
tetT	F：AAGGTTTATTATATAAAAGTG<br>R：AGGTGTATCTATGATATTTAC	169	40
*sul*1	F：CACCGGAAACATCGCTGCA<br>R：AAGTTCCGCCGCAAGGCT	158	60
*sul*2	F：CTCCGATGGAGGCCGGTAT<br>R：GGGAATGCCATCTGCCTTGA	190	60
sul3	F：TCCGTTCAGCGAATTGGTGCAG<br>R：TTCGTTCACGCCTTACACCAGC	128	55
sul4	F：TCAACGTCACTCCAGACAGC<br>R：CCGTCGGCTACAAATTGTTT	120	60
macB	F：GATACCGTTTACGAAATTGG<br>R：GAATCGAGACTTGAGTGTGC	364	58
*intI*1	F：GGCTTCGTGATGCCTGCTT<br>R：CATTCCTGGCCGTGGTTCT	146	55
*intI*2	F：GTTATTTTATTGCTGGGATTAGGC<br>R：TTTTACGCTGCTGTATGGTGC	164	55
Tn916/1545	F : GACAGTATTAAGCCATCAGAC<br>R : TCTTCCGAACACAATCATCT	142	50
IncQ *oriT*	F：TTCGCGCTCGTTGTTCTTCGAGC<br>R：GCCGTTAGGCCAGTTTCTCG	191	62
16S rRNA	F：CCTACGGGAGGCAGCAG<br>R：TTACCGCGGCTGCTGGCAC	193	60

1) R=A, G; Y=C, T; K=G, T; S=G, C; M=A, C; W=A, T; B=G, C, T; V=A, G, C; D=A, G, T; H=A, C, T; N=A, G, C, T. 2) The 10 μL reaction mixtures contain 5 μL ChamQ SYBR qPCR Master Mix (Vazyme, China), 0.2 μL each primer (Sangon Biotech (Shanghai) Co., Ltd., China), 3.6 μL ddH_2O, and 1 μL DNA template. 3) The thermocycling steps of qPCR: (1) 95 ℃, 3 min; (2) 95 ℃, 5 s; (3) annealing temperature, 30 s; (4) 72 ℃, 30 s; (5) plate read; (6) repeat steps (2) to (5) 40 more times; (7) melt-curve analysis, 60 ℃ – 95 ℃, 0.5 ℃ read. 4) The qPCR amplification efficiency is between 90 to 110% in all experiments.

Table S2. Summary of metagenomic sequencing, assembly, and ORF prediction.

Samples	Raw reads	Raw base (bp)	Clean reads	Clean base (bp)	Percent in raw reads (%)	Percent in raw bases (%)	ORFs
CK0d-1	45535984	6875933584	45156832	6809162188	99.16736	99.02891	930024
CK0d-2	45182870	6822613370	44818794	6758351987	99.19422	99.05811	924484
CK0d-3	52342670	7903743170	51912046	7827541507	99.1773	99.03588	1088253
CK2d-1	46863102	7076328402	46268060	6966893831	98.73025	98.45351	548461
CK2d-2	40753508	6153779708	40441878	6094676465	99.23533	99.03956	715460
CK2d-3	44529220	6723912220	44024130	6631065632	98.86571	98.61916	530313
CK8d-1	46966956	7092010356	46062170	6931928270	98.07357	97.74278	379202
CK8d-2	52546202	7934476502	51567796	7759542785	98.13801	97.79527	415449
CK8d-3	51566538	7786547238	50676118	7628875351	98.27326	97.97507	528970
CK16d-1	47802576	7218188976	47120412	7097112653	98.57296	98.32262	652049
CK16d-2	50062594	7559451694	49183852	7404020168	98.24471	97.94388	557154
CK16d-3	51964430	7846628930	51219496	7713984707	98.56645	98.30954	569594
CK24d-1	48684840	7351410840	48136708	7251239135	98.87412	98.63738	592569
CK24d-2	52854780	7981071780	52029156	7832569289	98.43794	98.13932	591542
CK24d-3	45448132	6862667932	44818696	6748937343	98.61505	98.34276	568247
GD0d-1	49028090	7403241590	48640768	7334451997	99.21	99.07082	1025284
GD0d-2	50170584	7575758184	49760396	7502779607	99.18241	99.03668	1073720
GD0d-3	39975314	6036272414	39652206	5978476630	99.19173	99.04253	861266
GD2d-1	44225750	6678088250	43799540	6601936318	99.03629	98.85967	651107
GD2d-2	45846020	6922749020	45188590	6805191791	98.566	98.30187	562809
GD2d-3	43711632	6600456432	43468998	6550861333	99.44492	99.24861	627625
GD8d-1	40955502	6184280802	40294918	6066646387	98.38707	98.09785	400456
GD8d-2	47560612	7181652412	46660580	7021874069	98.10761	97.77519	334224
GD8d-3	42055514	6350382614	41258820	6208461702	98.10561	97.76516	363057
GD16d-1	53165522	8027993822	52160622	7850393803	98.10987	97.78774	530832
GD16d-2	50216272	7582657072	49482926	7451874521	98.53962	98.27524	564626
GD16d-3	42744396	6454403796	42008250	6323797625	98.2778	97.97648	512439
GD24d-1	45983056	6943441456	45303374	6820291517	98.52189	98.22638	497898
GD24d-2	49619402	7492529702	48907066	7363705590	98.5644	98.28063	487540
GD24d-3	48448910	7315785410	47757424	7191133336	98.57275	98.29612	549367

Table S3. Individual adjR^2 of environmental factors for microbial community composition.

Environmental Factors	Environmental Factor Group	adjR^2 (CK)	adjR^2 (GD)
Cellulase	Lignocellulolytic enzyme activities (LEA)	0.04183	0.08093
Cellobiase		0.0615	0.08469
Laccase		0.12719	0.17668
Electrical conductivity (EC)	Physicochemical indicators (PCI)	0.09871	0.04267
Total nitrogen (TN)		0.22772	0.18443
N-NO_2^-		0.13044	0.10602
N-NH_4^+		0.15388	0.05216
N-NO_3^-		0.14141	0.12464
pH		0.11785	0.0521
Moisture Content (MC)		0.1731	0.10443
Temperature		0.39045	0.3072

Note: Environmental variables were checked for collinearity using variance inflation factors (VIF), and variables with VIF > 10 were excluded. The significance of each fraction was tested using 999 permutations.

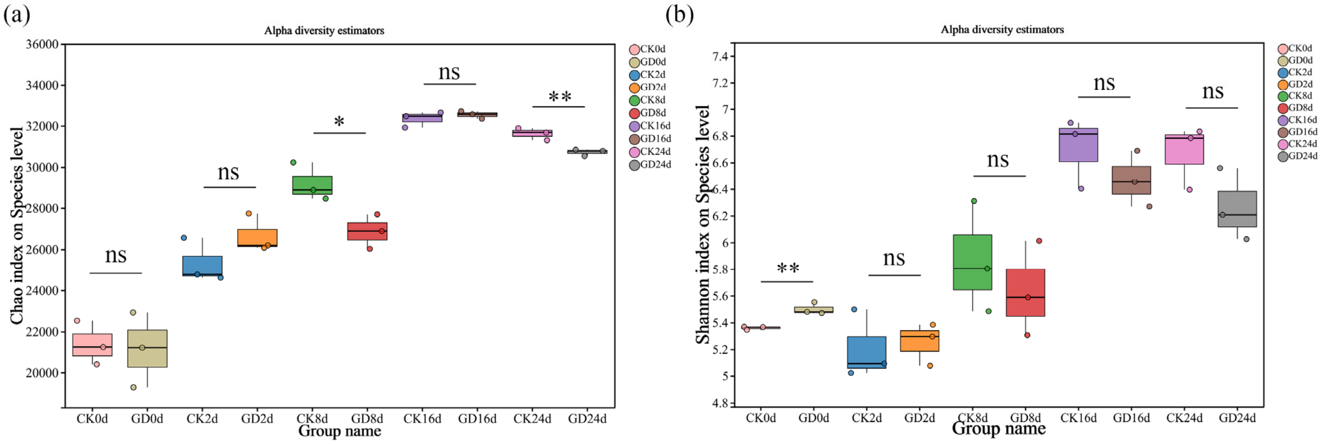

Figure S1. Dynamics of α-diversity in samples across different time periods. 'ns' represents no significance , $p > 0.05$, '*' represents $p < 0.05$, '**' represents $p < 0.01$.

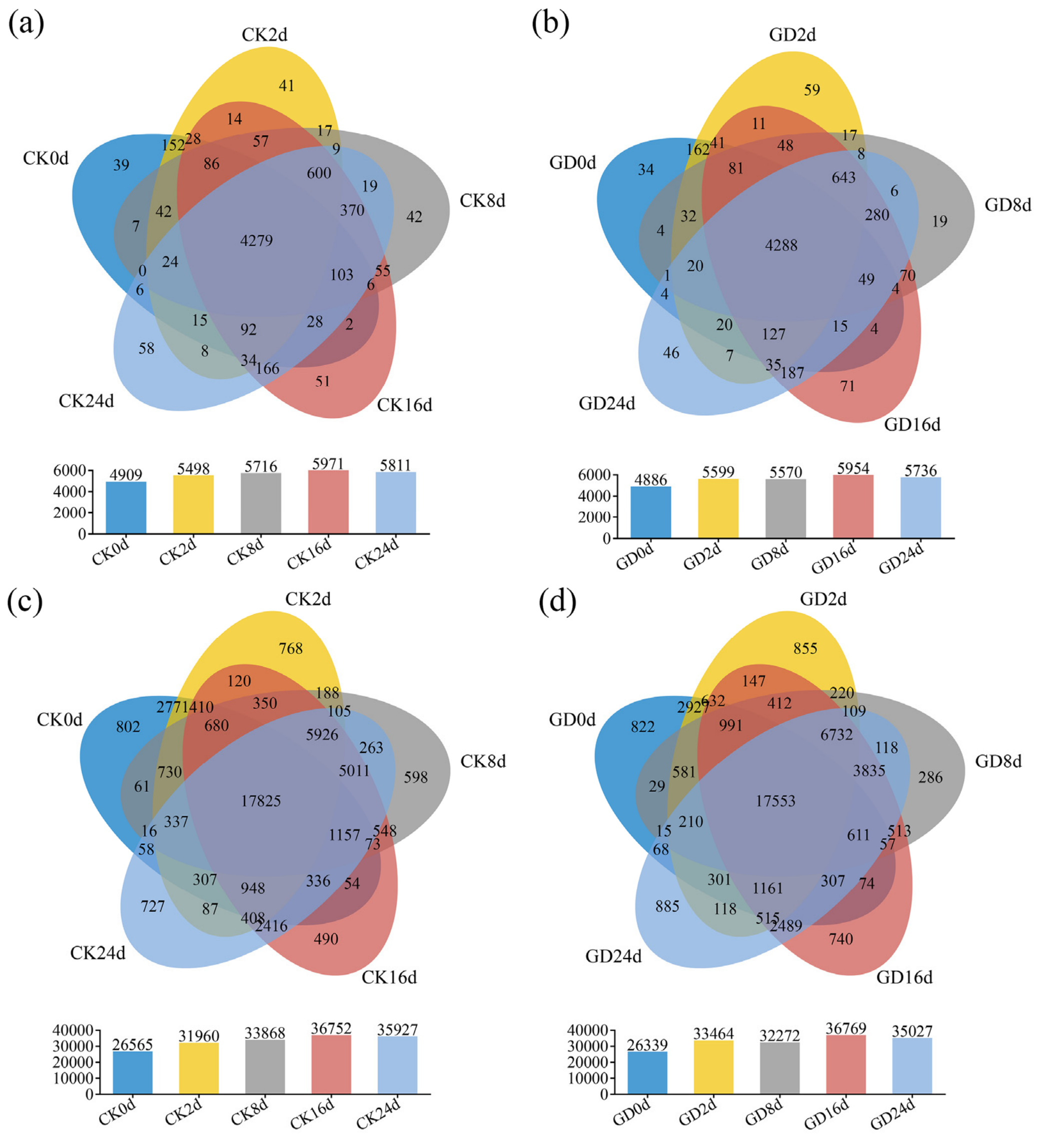

Figure S2. The venn diagrams of shared microorganisms at different time periods. (a, b) Genus-level venn diagrams for the CK and GD groups, respectively; (c, d) Species-level Venn diagrams for the CK and GD groups, respectively.

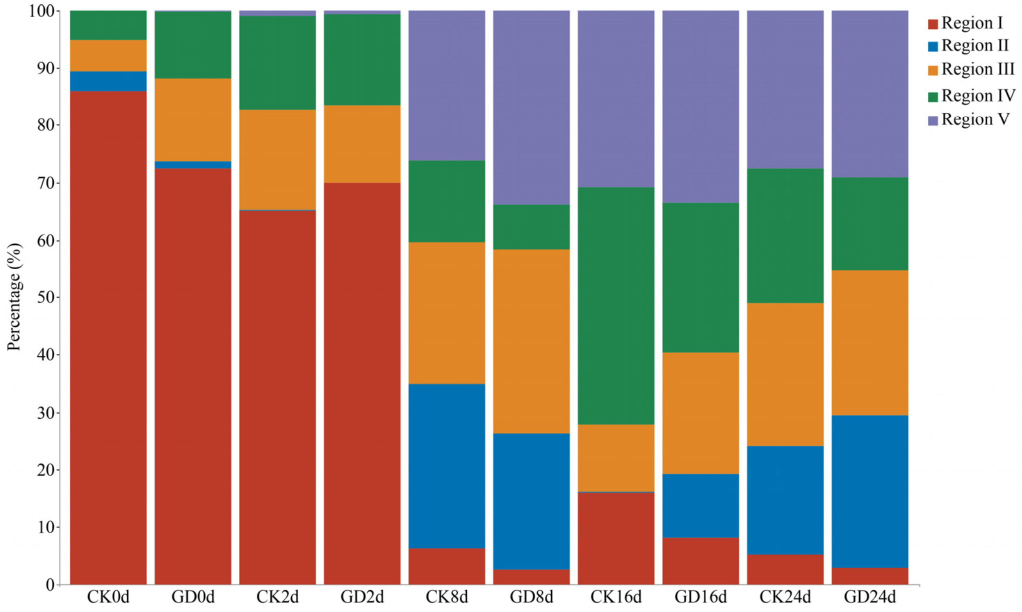

Figure S3. FRI ratio results in different regions of 3D-EEM. Regions I to V correspond to tyrosine substances (Ex/Em, 200~250 nm/200~330 nm), tryptophan (Ex/Em, 200~250 nm/330~380 nm), fulvic acids (Ex/Em, 200~250 nm/380~600 nm), soluble microbial by-products (Ex/Em, 250~500 nm/200~380 nm), and humic acid-like substances (Ex/Em, 250~500 nm/380~600 nm), respectively. Three repeats in CK and GD groups during day 0, 2, 8, 16, and 24.

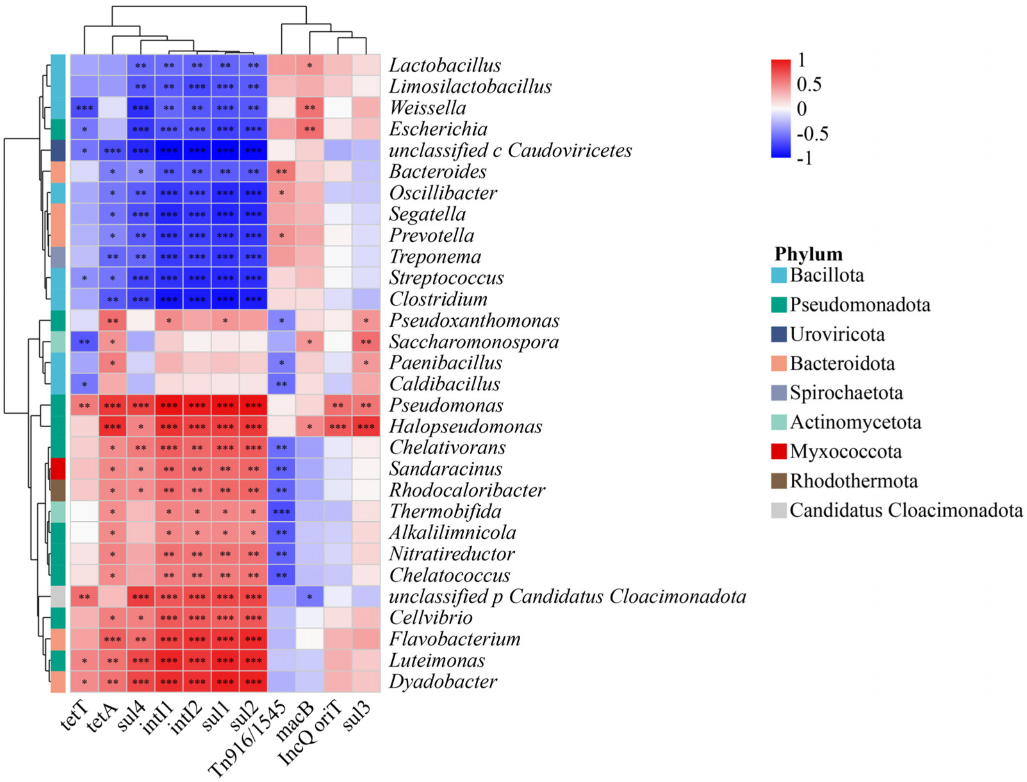

Figure S4. Correlation heatmap of ARGs and microorganisms classified at the genera level (Top 30). '*' represents $p < 0.05$, '**' represents $p < 0.01$, '***' represents $p < 0.001$.

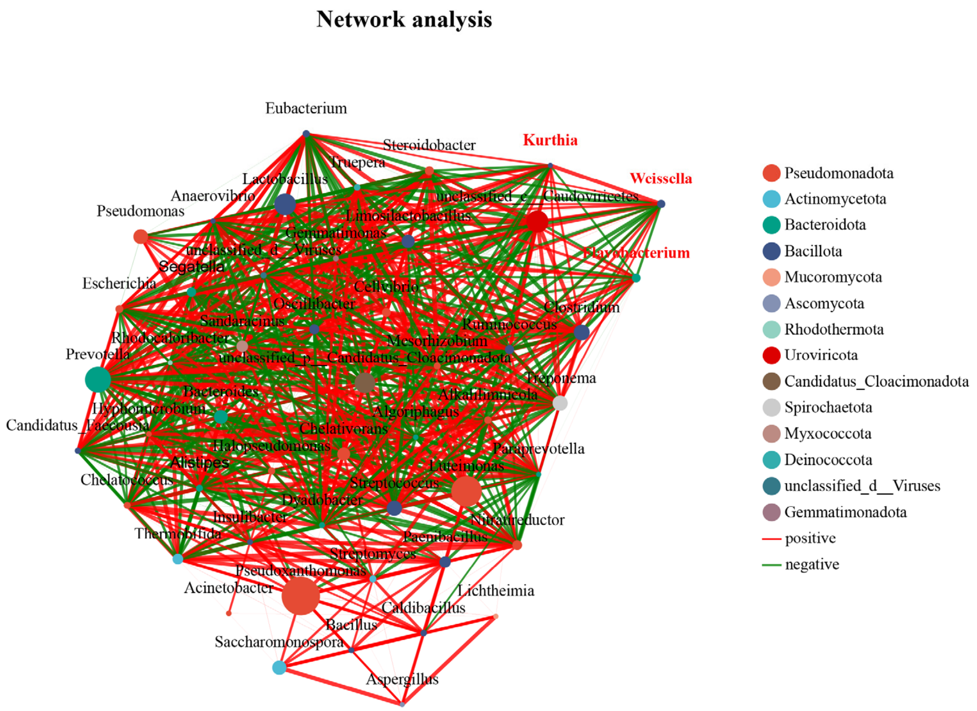

Figure S5. The network analysis of microbial correlations at the genus level.
